# A Consideration of Some of the Causes Which Give Rise to Dental Decay

**Published:** 1883-07

**Authors:** Alfred Carpenter


THE
AMERICAN JOURNAL
OF
DENTAL SCIENCE.

VOL. XVII. THIRD SERIES—JULY, 1883. NO. 3.

ARTICLE I.

### A CONSIDERATION OF SOME OF THE CAUSES WHICH GIVE RISE TO DENTAL DECAY.

A Paper read before the Odontological Society, November 6th, 1882.

BY ALFRED CARPENTER, M. D.

*From Transactions of Odontological Society, published in London Record.*

A study of the teeth among the lower animals is especially interesting to the searchers after first causes, and throws a flood of light upon development. Among some of the invertebratæ the teeth are clearly gastric, being a part of the stomach. In this position they do not depart from their epithelial character, for the mucous membrane of the abdominal tract is as epithelial as is the skin, and has the same origin. These teeth are composed of chitine impregnated with lime and salica. The teeth of the sea urchin are made up of prisms of carbonate of lime, with leaflets interspersed between the sprisms, and in them we find a fine plexus of anastomosing canaliculi. It is among the shells of the mollusks that we first find a division into an outer prismatic, or fibrous, and an inner laminated tissue, similar to that in vertebrate teeth. Among the terebratulæ we find (according to Dr. W. B. Carpenter) tubes traversing the substance, forming branched horizontal channels, sometimes connected with rounded cavities, which seem to be analogous to the lacunæ and canaliculi of the *crusta petrosa*. The shell of the mollusk, which has become calcified, corresponds to the epidermis of the higher animals, and thus teeth have the same origin as those of the vertebratæ, viz., being epithelial. The prism-like appearance is due to the cellular structure and the transverse striæ corresponding to the thickenings of the cellular membranes, where the layers come into contact with one another. My great namesake states that in his opinion the calcareous network which is the shells of the invertebratæ generally, corresponds to the fibrous structure of the cutis of the higher animals, and which has obtained a calcified condition.

Let us now ask a question or two as to epithelium, and the duties it has to perform.

1st. It is protective; of that there can be no doubt.

2nd. It is excretory.

That which applies to the whole may apply to any part.

The nails and the hair undoubtedly epithelial, and equally liable to changes from which those organs spring. There is difference, however, between the teeth on the one hand, and the hair and nails on the other, inasmuch as the latter are made up principally of organic substances, and are of a horny nature which is almost indestructible; it is allied to, but slightly different from gelatine, and is sometimes styled keratin. This material therefore occupies the position of indestructibility which is held by the enamel of the teeth, but which in the latter case is mainly due to inorganic matter. Its chemical character is not clearly set out, but whilst enamel has a minute portion of organic matter in its composition horn and nail have but little inorganic deposit, perhaps even less of inorganic matter than enamel possesses of the organic. There is thus a curious though antagonistic analogy, minute quantity of organic matter in the structure of enamel is the channel by which the life of the tooth is kept up; and a dead tooth, like a dead shell, is somewhat different to one that is still in direct and living contact with the body of its owner. A necrosed tooth will easily take up decay. If an expert examines the collection of a conchologist, he will be able without hesitation to point out in the collection those shells whose original bearers had died a natural death (and which had been afterwards exposed to external influence as a sequence to that death), from those which had been taken alive. A shell picked up on the sea-shore with out a living inhabitant is quite different to those which are taken with the animal living in them, and which kind alone make fit addition to the collector's drawers. Change take very rapidly in the one kind of shell which do not show themselves at all in the other. The organic matter of the living shell is a reality, and it lives with the animal itself. The changes which take place in the dead shell are rapidly promoted by friction, by the action of the air, and by acting upon the organic matter which, being deprived of its vitality, allows of alterations taking place in it to which it was able to furnish a complete barrier when in the living state, and in absolute union with the living animal, although it is not permeated by blood vessels, and there is no evidence of nerve tissue in its substance. The friction on the sea-shore is not greater and the action of salt water is not different upon the dead shell to that which it has upon the living shell and animal together. Yet these actions are operative on the dead shell in the one case, and are without injurious effects in the other. The only difference between the two shells is that of the vitality of the organic matter in the one case, and its absence in the other.

Suppose we minutely examine the oyster-shell. Sections of this shell, when carefully prepared, make very pretty miscroscopical objects, and show markings which correspond very closely with the lacunæ and canaliculi of the *crusta petrosa* of the human teeth. Oyster-shells consist mainly of carbonate, not of phosphate of lime; the phosphorus is retained by the animal, and it does not become an excretion in the shell; it is, however, to be found in the constituents of the tissues. The phosphate, not carbonate, is the principal inorganic ingredient of the human tooth. There is here, then, a manifest difference between the excretion which forms the shell of the mollusk and that which forms the tooth of the vertebrate animal. You may ask what I mean by this expression of excretion. It is simply the statement of a fact that every deposit which is made by any organ in the body is an excretion as far as the rest of the body is concerned. The deposit is able at times to destroy the life of the rest if it be kept in contact with the body at the wrong time. That whether it is urea, which is the organic salt excreted by the kidneys, or the carbonic acid thrown off by the lungs, and the cholesterine which the liver gets rid of, and which is probably one of the products of nerve action; the epithelium, which is shaken out of one's flannel shirt when the latter has been worn too long, or even any secretion such as milk or saliva, these are all excretions, and once formed they must not be retained in the organ or cells which have formed them, or mischief will result to that organ. The hair and nails are the manifestation of an excretion; the materials which form those parts, and which are intended as defensive organs, having been used up in some special work about the body, are no longer required within the precincts of that body, and being done with, the materials which form those appendages are exuded from the body. The materials of which the shell of a mollusk is formed are in the same category as those of the nails and hair of vertebrate animals. They are done with as far as the nutrition of the active organs of the body is concerned, and they become excretions, and at the same time are made into protective organs. The phosphate of lime of which the bones, and especially the teeth, of vertebrate animals is formed, is in the same position, for it is an excretion. It is done with, and ordinarily it cannot again enter the circulation and be used up for any other purpose. There are some exceptions to this rule which allow of the excreted material being altered in its chemical character when required for admixture with other extraneous matter, and then it may be absorbed again for immediate use and ultimate removal; but as a whole, the rule is as stated. An excretion is a poison as far as the rest of the body is concerned. If that excretion is suddenly checked, and the materials which from it are thrown back again into the animal economy, mischief will result, and disease be set up. With regard to some secretions, the quantity is comparatively unlimited, but with others it is not so, the quantity is small and cannot be largely increased. We have instances of abnormal growths, such as Mr. Darwin alludes to in his work on "Animals and Plants under Domestication," in which there is a notice of the relation which holds between the abnormal growth of hair as compared with teeth; it sometimes happening that excessive development of hair has been attended by an edentulous state, as if nature could not form more than a certain quantity of some material, and if one is formed in excess the is a deficiency of there other. Two men were shown in Paris some years ago, who were father and son. They were without teeth, but with their faces and necks completely covered with shaggy hair, like unto Scotch terriers. It seemed as if in these cases the organic matter required for the hair was provided at the expense of the phosphate of lime; or perhaps the absence of the latter excretion allowed the hairy development, and then the organic matter which was required for food development could not be diverted back to the dental tissue, or was not at hand at the moment at which it was wanted.

If the organism is defective in its constitutional parts at its first foundation—if the products necessary for first development are wanting, we may have defect in the first lines of life, and hence a tendency to decay may originate at the first moment of our birth, and may we say, with Pope—

"That man,
The moment of his breath,
Receives the lurking principle of death;
The young disease, which must subdue at length,
Grows with his growths, and strengthens with his strength."

It has been fully recognized that phosphate of lime is the principle organic element in the chemical constituents of the teeth; it also forms a large part of all bony matter; it has been assumed that the formation of bone is the main purpose for which the phosphates are required. I am bold however, to think that this is clearly not so. Phosphate of lime is a constituent of all organic life, or at least, some salt of which phosphate of lime is the result. It is probably the sequence of the act of living. Some portion of it plays an active physiological part in the manifestation of vital phenomena, and no life can be carried on without it, either as an active part or as a result of action. It is the principal inorganic matter which is first used up in the progress of development, and there must be depots for the used-up material. There exists no tissue in animated nature which does not contain phosphate of lime in some form or other as the sequence of the activity of that tissue. It has an influence on organic life; possibly in the more soluble form of superphosphate, and its presence is most likely absolutely necessary for the changes which take place in cell life. It is known that it exists largely in nerve matter. That phosphorus, or some salt of phosphorus, in some form or other, is indispensable for nerve development; and it is probably, therefore, that those bodies which are placed low in the scales of creation, and which do not possess a distinct nervous system, have a certain power, resulting from the presence of a salt of phosphorus, which enables them to carry on their nutritive and other necessary actions without the intervention of a brain, or spinal cord, or even a nerve ganglion of any kind. The presence of some salt of phosphorus is absolutely indispensable for the formation and continuance of cell growth; and in the production of new cell life the phosphate is formed, and after a time has to be removed from its position in the animal economy. It becomes an excretion, and has to be removed from the tissues of the animal. Even before a regular nervous system is formed, and in the early days of organic life, we find its exudation prepared for by the epithelial structures.

Phosphorus is present in the lowest forms of vegetable life as well as in those which are animal. It exists in an appreciable quantity wherever there are fibres and cells. It is found as a necessary constituent in our daily food. It is abaudant in milk, in flesh, and in wheat. The more active the process of development which is going on, the more do the phosphates in some form or other abound. The more active the process of development is going on, the larger is the quantity of phosphorus in the organic matter of the specimen under examination. It has been shown that wheat grown in a soil completely deprived of phosphates can grow into a plant but cannot produce grain. There is a great difference, an antagonism, indeed, between animal and vegetable life in this matter, so that phosphates are not excreted as if they were used-up material by plants, but they are laid up principally as a store in the seed of the plant for future use. The phosphate does not form an intrinsic part of the skeleton of the plant, but it accompanies the albumenoid substance whose existence is anatomically independent of the solid tissues. It appears to be a special part of the work of the plant to secrete this in the material which it prepares for the norishment of animals, who feed upon the result and are able to carry on their own functions by its means. There is an important distinction, therefore, between the position of the phosphates in the one as compared with the other; and it corresponds to some extent to the position occupied by carbon and oxygen in the two kingdoms. The latter element is, as you know, excreted by the plant as the result of its organic life, the carbon being retained and used in building up the tissues of the plant; but in the animal life it is the carbon which is excreted as carbonic acid, the oxygen being exchanged for it. The phosphorus is in combination with the organic, not the inorganic parts of the body. The largèr the amount of phosphoric acid in a given quantity of organic vegetable matter, the more vigorous are the actions which are going on, and the greater is the vitality at work in the material under examination—not, however, for the purpose of forming a skeleton. I wish you to bear in mind that it is in the albumenoid material, and not in the skeletons of plants, that phosphoric acid, or some salt of which phosphorus is the base, is most abundant, and that most plants cannot perfect their species and prepare seed for future developments if phosphorus is withheld. There is also a point in which there is no antagonism between plants and animals, which is worthy of notice as bearing directly upon our subject. There is a special liability to disease among plants as well as animals under certain conditions. The epidermis of plants which are not sufficiently supplied with phosphoric acid is prone to suffer from disease, and a wheat-field in which the soil no longer contains phosphate salts is very liable to blight, to rust, and to mildew. There is a kind of defective nerve power, so to speak in the structure, which lays the plant more open to the attack of outside enemies; whilst there is absolute failure in its ulterior development, it cannot perfect its epidermis properly. If there is no phosphate in the soil at all, the plant will grow as long as there is phosphorus in the seed itself but no longer; as soon as this reserve is used up the nerve force is lost and the plant decays. Can we not, in this view of the matter which we obtain from the vegetable kingdom draw some inference which may be of service in considering the cause of decay of teeth in animated nature? If phosphorus, in some form or other, is not provided in the food we eat, it is found by everyday experience that the constitution suffers exceedingly. There are conditions of life in which it is not so provided. In some of the great cities of the Empire there is, or was, a great deficiency in the natural food which infants require for proper growth. In those places rickets (or rachits) abounds. This is a disease of bone in which the phosphate of lime is very deficient: the bones are soft and cellular; the teeth are also defective, and the intellect inferior from inertness of cerebral action.

I am of the opinion that this state is brought about by a deficient supply of proper pabulum for natural development.

The child is not nourished by its own mother's milk, and cow's milk cannot be obtained in sufficient quantity and quality by the poor people in such cities ; they either do not get it at all, or if they do get it, it has been watered, or it is supposed by the ignorant parents that something else is better for them. They do not get fresh vegetables, which contain phosphate in the soluble form, and they are supplied with farinaceous food, in which the larger portion of the cuticle which contains much of the phosphoric salt has been removed by decortication. The result of this kind of feeding is that the nervous system does not get its proper supply for healthy developments, and as a sequence, those organs which are dependent for their own development upon the composition which results from the act of brain cell growth, cannot be perfected. The teeth fail in their proper construction ; there is more organic than inorganic matter in their structure, and, like the proper skeleton, they cannot perform the functions which nature intended that they should do. A purely flesh diet will produce rickets, and lead therefrom to teeth failure.

On the other hand, it has been noted that if phosphorus is given in excess of the requirements of the system, as has been done sometimes by experiment, it will itself set up mischief in the bones, especially if the lime salts are withdrawn and it is found that in those animals in which rickets have been thus produced, another material called lactic acid is found in excess in their structure and in their excretions. If lactic acid, such as usually developes in milk which has been kept too long, be given as a food, and which the poor children in our great cities too often get every day when they get milk at all, while phosphorus and lime salts are withheld, rickets, or at least a soft state of bone tissue, is rapidly developed.

In some large cities, such as Glasgow for instance, it happens that lime is not a constituent of the water supply. Children therefore run the chance, in that and similar cities of having no lime provided in the water, whilst no phosphate is at hand such as milk and vegetables give ; they ignore the advantages which oatmeal supplies, and which Scothmen as a rule think much of, and as a consequence, rickets and its allies are common diseases.

There is another disease which used to be very common in the naval service of this country in the last century, viz., scurvy. Two centuries ago it was a complete scourge in all northern Europe, as well as in our own marine service, but the discovery of its cause has materially diminished its incidence, though it is even now in our own day much more frquent than it should be, and its effects are not always recognized in the minor form which it does even now appear amongst us, both in the sick room, and also among poor people in thickly peopled districts.

Its first incidence is upon the gums ; the teeth loosen and decay ; there is a tendency to hemorrhages and effusions of blood from mucous membrane ; the slightest injury to the skin produces a bruised appearance. The cause for this condition, in my opinion, is the absence of fruit and vegetables from the common diet of the sufferers. The effect of fresh fruit is to diminish those acids which result from the consumption of a flesh food, which is too highly nitrogenous. The alkaline vegetable salts which fresh fruits contain become decomposed into carbonates, which pass off with the urine, and take with them, or assist to discharge by other means, some of the nitrogenous matter which exists as *debris*, which would, if left there, from lithic acid and its allies. The small quantity of a phosphate salt which they contain is used up in nerve nourishment, and a phosphate of lime is prepared. Fruit and vegetables are extremely advantageous, therefore, in those conditions of the system in which there is a lithic acid diathesis. They prevent the formation of urate of soda, which often by its deposition in the peridental membrane induces those diseases of the teeth to which the gouty classes are especially liable. Improper food in infant life does lay the foundation of disease in the dental appendages which, following upon those produce by hereditary causes, are very serious in their result. So improper feeding in our latter days will do the same. I sometime meet people who never touch fruit or vegetables in their daily diet. They are on the high road to suffer from those diseases which result from diet which is too nitrogenous—such diseases as gout, fatty degeneration, scurvy. They are depriving themselves of the pabulum which is necessary for the proper nourishment of both nerve and bone, and are keeping within the precincts of their body material which ought not to be there. They either have lost or will lose their teeth sooner than other people, simply because the supply of material necessary for maintenance is not afforded. I sometimes meet with sick persons who have bleeding gums, continuous neuralgia, many carious teeth, and a tendency to hemorrhage produced by the slightest injury, who never touch fruit, and seldom eat vegetables, because they believe that those articles disagree with them, and, as they suppose, set up dyspeptic and other gouty complaints—just as some people will not allow the housemaid to dust their libraries and book-shelves because of the dust they are likely to kick up, and the displacements which are sure to follow. Is it a wonder that dust and dirt abound in that library, and *debris* of a wrong character finds a settlement in those positions in which such *debris* is out of place? It is just so in the human economy. If the diet is not a proper kind, if some elements are ignored altogether which nature intended us to take, there is sure, sooner or later, to be a defect in our excretion and the fundamental condition of the body is altered. It is posible that the teeth are the depots for the deposit of the phosphate of lime, which is formed in very minute quantity in the first months of human life, and before the bones are ready to receive it, it may be the *debris* of the first cells which form in the infant. Let there be some defect in innervation as may arise in the progeny of a syphilitic individual and we may see the result in the defective character of the phosphate of lime which is deposited in the cells of the ivory and the enamel membrane of the new development. It is not a pure salt; it contains something else besides phosphate, and, as a result, the cells themselves are imperfect, both in shape and number; the striæ upon them are unerring witnesses of the taint which the father has inflicted on his own progeny, and which taint will probably show itself later on in the life of the child, by defective nerve centers, by a mental weakness, or, it may be, by paralysis, or by insanity, and other nerve disorders, which will require much care to prevent their development, even if they can be prevented.

The dentist has a serious duty to perform when these cases come to his notice, viz., to inform the parent of the constitutional taint which exists in the child, and to advise that measure be taken to diminish its incidence, by counteracting its influence by such means as temperance, chastity, and correct diet. The children of this class are more prone to nerve disorder than other people, those nerve disorders often being of the most painful kind. A warning to the parents, and, if the child is old enough, a warning to himself, that there is very much to be done to get rid of a taint which touches the foundation of life, and effects the battery before it can show its results in the *debris* of that battery. How this effect is first produce is unknown to us, but that it does show itself in the second and even a third generation is now fully acknowledge, and it ought to be taught to young men as one more argument against the vice of unchastity and the danger of uncleanliness. The sequence is probably due to failure of nerve growth; the teeth are sometimes shed even before they are fully formed. The teeth cannot develope, because the material required for their production is not properly formed; there is no *debris* of the right kind.

Caries also is properly favored by the condition which promotes tubercular tendencies. A minute particle of perverted protoplasm is laid down in the cell membrane which forms the dentine. It is chemically defective from having a tendency to fatty degeneration rather than to a proper deposit of phosphate salt. A single cell may be thus impaired by the defective structure, or a half-dozen cells may be touched. As time goes on the defective cells soften, and, by means of the imperfection of the organic matter which pervades the tooth, which is in excess of its requirements, there is a want of proper balance between the enamel, dentine, and the organic matter; there is defectiveness in their composition as well as in the support which is required, and, in consequence, the tooth is more liable to wear and tear than would have been the case if the inherited defects had not existed; a small hole is punched in the tooth, which if not resisted by the dentist, will give a *locus standi* to foreign bodies, to vibrios and to bacteria, and thus allow of assaults being made upon tissue which is still sound by the secretions which the living organism produce in the cavity of the tooth, and caries than arises.

An attempt is generally made, though unsuccessfully by nature, if not assisted by art, to remedy the mischief. A small amount of chemical action takes place in the hole, which failing in its object in the course of time leads to extension into the pulp cavity, and ultimately to the total loss of the tooth. It is necessary for the best interests of the children of tubercular parents that their teeth should be frequently inspected by the dental surgeon, and those punch-like holes cleared and stopped before the pulp cavity is reached. In these cases the conservative power of the dental surgeon is very manifest. He should advise a close attention to the rules which obvious tendencies to tubercle require to be observed. He should urge upon the parent of such children to beware of the mischief which, unless due care is exercised, will be in store for them. If germs of perverted protoplasm are found in the dental organs they will be found in other tissues. I have reason, however, to believe, that when such tendencies show themselves on epithelial structures in early life, and when the deeper seated organs are not at the same time affected, that there is a greater chance for hereditary conditions to be overcome. The tendencies to disease being limited to epithelial tissues, it is reasonable to hope that, with due attention to the rules which should be observed for securing a healthy state of body, that there will be an evacuation of *materies morbi*, and so the child may escape from the evils which it has inherited.

I have seen in some families, where the syphilitic taint has been undoubted, that those children with defective teeth who have skin eruptions, in whom the defect has been recognized, and all exciting cause for further mischief withheld, such as the abuse of mercurial medicines and excessive use of animal diet, that they have escaped the evils which have befallen other members of the family in whom the epithelial structures have not been at first affected, but in whom cerebral mischief has shown itself at an early date. I have seldom seen the presence of tertiary syphilis on young children without finding evidence of mischief in the teeth, the hair, the nails, the epidermis, all more or less showing the taint. In other instances I have also seen the teeth alone giving such evidence, and in those cases the individuals have grown up to man's estate in fairly good health, though the chances of such results are not great. But if, in addition to the taint, as shown by the epidermis, there is also some organic disease in some splanchnic viscera, there is no likelihood that the child will make old bones, or even grow to man's estate. A large number of those cases in which early death arise from cerebral disease are closely connected with hereditary syphilitic taint, and the insight as to cause is obtained from the irregular, the jagged, and stained condition of the dental organs. We get a similar insight into the tubercular tendencies in certain families. The teeth are not irregular, jagged, and stained, but they are thoroughly carious—there is a want of true bone, of true enamel. The material provided is fatty and friable, and with this state of the teeth we have generally a tendency to bow legs and soft, irregular skulls. In these cases it is defective innervation from the first, which has arisen from foul air, deficient sun-light, and bad food. All these causes have been at work upon the organization of the parents, and show themselves in their progeny. A continuance of such causes acting upon the child increases very much the tubercular tendencies which exist in the constitution, and render more easily liable to be influenced by cacozymes or septic bacteria, which are more easily developed, and flourish most in foul air and dimly-lighted dwellings. Children, therefore, having the first set of teeth carious, require something more than surgical aid. The parents should be advised to avoid the causes which promote septic developments, to cultivate the benefits which are to be derived from the beneficial influence of sunlight, from a pure dry air, and from simple, unstimalating food ; and the surgeon who only attend to the teeth has only done a part of his duty ; but it is also only fair to say that this class of cases seldom comes into the charge of the surgeon dentist at all.

I have seen conditions in strumous children similar to those born of syphilitic parents. When the evil has fallen upon the epidermis, and has shown itself in defective teeth with scaly or vesicular eruptions upon the skin, the internal organs have escaped, and the children, having thrown out the morbid matter which they inherited, have grown up into healthy and well-developed individuals ; this is, provided the laws of health have been observed, good, unstimulating food fresh milk and vegetables used, and fresh, dry air with sunlight, sought for. I have, on three or four occasions, seen two or three children in the same family with bad temporary teeth and exzematous eruptions, yet, grew up satisfactorily whilst those with clear skins, and not showing any defects in the epidermis, have died in early life of cerebral disorders or mesenteric diseases of a tubercular character.

The effects of the gouty diathesis do not show themselves so markedly upon the teeth in early life, as the case with the syphilitic and the tubercular states. The tendency to disease which this diathesis does promote among the descendants of gouty people is more likely to manifest itself in another way, and other tissues suffer more than the teeth, though caries is not uncommon. The quality of the nerve cell is not necessarily damaged in a gouty man, and, as a consequence, the teeth do not often suffer. The children of gouty people experience tendencies to blood maladies which (just as syphilis in the beginning of life) affect in the end every organ of the body ; but it is connection with the nitrogenous material, and not with the phosphorus of the constitution—the organic rather than the intellectual regions of the suffer from its incidence.

The rise of gout and its sequence are connected with excess of material, and with derangement of the digestive organs, which is the consequence of that excess, rather than to the introduction of an extraneous poison, or by the action of an arrested growth. Syphilitic taint is probably due to a parasitic life ; tubercular growth arise from the presence of perverted protoplasm in the blood, in which bacterial or other forms of parasitic life can increase and multiply. Syphilitic conditions are due to a taint which will affect every person into whose organization its first cause finds admission, and from whose tissue it has not been immediately ousted. The tubercular will only develop in those person who have lived unhealthy lives ; its influence is continued by their surroundings ;whilst gout is not brought on by either of these conditions. It is a state induced by excess, and its sequences are on different lines. The surroundings may be quite healty, but the habits are vicious, indolent and self-indulgent.

The condition which produced it are not common to, and are not obtained in early life. They more often arise after the man has attained his full growth. His progeny are born into the world, in the majority of cases before the humors of the body have been able to act injuriously upon the sexual organs. It is seldom that a constitution is thoroughly gouty before a man is forty or fifty years of age. Thoroughly gouty men do not have families, to any extent after they have become saturated with gout; thus the specimens afforded us are limited, as far as children are concerned. Still it is, I think, a fact that the teeth do decay sooner in some of those who are descended from gouty parents : they are more subject to caries, which disease has its origin in fatty degeneration of the dental cells, and the subject of it especially liable to those peridental inflammations which ultimate lead to necrosis. Gouty men and women also suffer very much from neuralgia and other conditions of the system which have their origin in a lithic acid diathesis, and they can only be properly eured by associating the dental treatment with a proper deit, and the judicious use of remedies against the gouty tendency. The presence of a lithic acid diathesis is accompanied by pain, whlch reflects itself upon every function which the body has to perform. Indeed, the state of the mouth is frequently the nemesis which attends upon over-indulgence; for the tender state of the gums and teeth put an end to feasting, and induces that fast which is the true cure for excess of nitrogenous material in the blood.

[TO BE CONTINUED]

ARTICLE II.

